# Factors influencing the decision of Nigerian optometry students to work in rural areas upon graduation: a cross-sectional survey

**DOI:** 10.1186/s12909-024-06281-6

**Published:** 2024-11-21

**Authors:** George Nnamdi Atuanya, Babatunde Ismail Bale, Emmanuel Ebuka Elebesunu, Alhaji Umar Sow

**Affiliations:** 1 Department of Optometry, Faculty of Life Science, University of Benin, Benin City, Nigeria; 2 Department of Medical Laboratory Sciences, Faculty of Health Sciences and Technology, University of Nigeria, Enugu Campus, Enugu, Nigeria; 3 College of Medicine and Allied Health Sciences, University of Sierra Leone, Freetown, Sierra Leone

**Keywords:** Nigerian optometry students, Rural area, Urban area, Education, Health workforce, Eye care

## Abstract

**Background:**

Access to eye care in rural Nigeria remains limited, as most optometrists work in urban areas. This study explores the factors influencing Nigerian optometry students’ decision to work in rural settings after graduation.

**Methods:**

A cross-sectional survey was conducted among 400 optometry students from ten accredited Nigerian universities. The students were surveyed on their preferences regarding rural practice and the factors affecting their decisions.

**Results:**

The majority of respondents (81.3%) were not inclined to establish their first optometric practice in rural areas, with poor living conditions (26.34%) being the most common deterrent. However, a significant proportion (52.8%) expressed willingness to consider establishing subsequent practices in rural areas. Motivation to help the community (56.6%) and the potential to enhance their optometric practice (74.6%) were key drivers for rural practice. Chi-square test revealed that participants’ year of study had a significant influence on their preference to practice in rural areas (*p* < 0.05). However, there was no significant connection between participants’ gender and place of origin, and their preference for rural practice (*p* > 0.05).

**Conclusion:**

While many students, particularly from urban backgrounds, are reluctant to initiate practice in rural areas after graduation, primarily due to concerns over living conditions. In contrast, students from rural backgrounds show a higher likelihood of considering rural practice, especially within NGOs or the public sector. Hence, such factors should be considered by academic institutions and government bodies when designing policies to address workforce imbalances.

## Background

In numerous regions across the globe, inadequate access to healthcare services, particularly in rural areas, contributes to poor health outcomes [1, 2]. Rural healthcare disparities manifest in various ways, including the uneven distribution of healthcare personnel, thereby affecting healthcare delivery in rural communities [3]. Consequently, people residing in rural areas should not be marginalized when it comes to healthcare coverage, including eye care.

Regrettably, the ratio of optometrists to the population is remarkably low, standing at 1:101,146 in Western Sub-Saharan Africa and 1:71,000 in Nigeria [4]. This falls considerably short of the World Health Organization’s (WHO) recommended target of one optometrist for every 50,000 people [4]. Moreover, the majority of eye care providers in Nigeria are concentrated in urban areas [5]. Over 80% of the 4,000 registered optometrists in Nigeria practice privately [6], exacerbating the unavailability of eye care providers in rural and remote areas [7].

To mitigate the burden of visual impairment and blindness, concerted efforts are required to bolster and maintain the optometric workforce in rural and remote areas [4]. This would ensure that an adequate number of optometrists and other eye care professionals are available to meet the healthcare needs of these underserved regions.

Comparable investigations have been carried out in South Africa [8], Ghana [9], and India [10]. These studies revealed that students originating from rural backgrounds expressed a desire to establish their initial and subsequent practices in rural areas. In contrast, students from urban backgrounds displayed this inclination mainly in India, with varying motivating factors drawing them to such choices, irrespective of their origins.

Eye and vision issues have been noted as more common in rural areas compared to urban settings [11], and a substantial portion (100.5 million) of Nigeria’s population resides in these rural regions [12]. In many developing nations, including Nigeria, there exists an imbalanced distribution of eye healthcare professionals, which disproportionately affects rural areas [9]. This is primarily due to the concentration of a majority of eye care practitioners in urban areas, leaving rural residents, who often require their services the most, underserved [7, 13]. Hence, it becomes crucial to explore the factors that impact the choices made by undergraduate optometry students in Nigeria when it comes to selecting rural communities for their practice. This is of utmost importance because optometrists, in collaboration with other ophthalmic professionals, play a vital role in providing eye care services to underserved regions.

Consequently, this study contributes to the existing body of knowledge on healthcare workforce distribution by providing new insights into the factors influencing Nigerian optometry students’ willingness to work in rural areas. It complements previous studies conducted in South Africa, Ghana, and India, focusing specifically on the Nigerian context. It will serve as a resource for policymakers aiming to improve the availability of eye care services in underserved rural regions of Nigeria by addressing the challenges related to optometry workforce distribution.

Additionally, while optometrists are recognized as the primary eye care providers in Nigeria, it is crucial to highlight the distribution of other eye care professionals, such as ophthalmologists. According to recent reports, the majority of ophthalmologists are concentrated in urban areas, further exacerbating the disparity in rural healthcare services [14, 15]. This imbalance underscores the urgent need for targeted interventions to ensure equitable access to eye care services in rural Nigeria​​. Therefore, the primary objective of this research is to comprehensively investigate the intricate factors that influence the decisions of Nigerian optometry students to pursue careers in rural areas following their graduation.

## Methods

### Study design

**Fig. 1:**
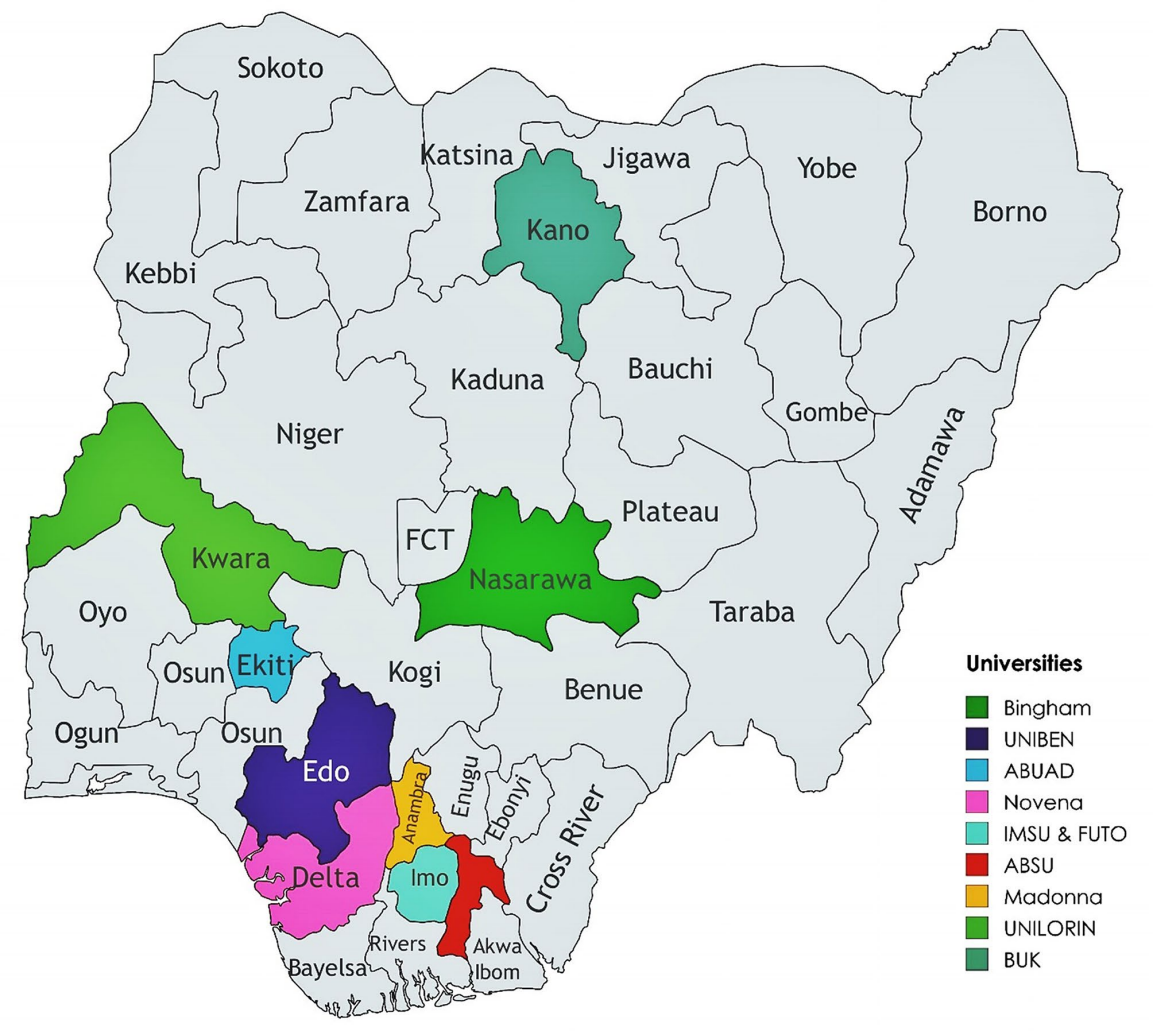
Geographical location of accredited Universities for Optometry in Nigeria [16]

The study was a cross-sectional study and a convenient sampling technique among optometry students in ten accredited Nigerian universities that offer the O.D programme. They include; University of Benin (UNIBEN), University of Ilorin (UNILORIN), Bayero University, Kano (BUK), Abia State University (ABSU), Imo State University (IMSU), Federal University of Technology, Owerri (FUTO), Bingham University (BU), Afe Babalola University, Ado-Ekiti (ABUAD), Novena University (NU), and Madonna University, Elele (MUE) (Fig. 1). This study encompassed optometry students from the undergraduate level, spanning from first-year to sixth-year, who were attending one of the chosen universities during the 2021/2022 academic year. The study took place within the timeframe of June to August in 2023.

### Study sampling

No existing literature provides a verified population of optometry students in Nigeria. Prior to this study, the current (2021/2022 academic session) total population of Nigerian optometry students from our study location was calculated to be 3,419. This was obtained with permission from the National and University chapters of the Nigerian Optometric Students’ Association (NOSA), based on the official departmental class lists across all available years of study. The sample size for this study was determined using Cochran’s formula [17], which is appropriate for cross-sectional studies with large populations. The formula is outlined below:

$$\:n=\frac{{Z}^{2}\:\times\:\:p\:(1-p)}{{e}^{2}}$$

where:

*n* is the sample size.

*Z* is the Z-score corresponding to the desired confidence level (1.96 for 95% confidence).

*p* is the estimated proportion of the population with the characteristic of interest (assumed to be 50% for maximum variability) [18], and.

*e* is the margin of error (set at 5%).

Therefore, we calculated a minimum sample size of 345 participants. To account for potential non-response or incomplete data, a 10% attrition factor was added, resulting in a target sample size of approximately 384 participants. Ultimately, we obtained responses from 400 participants, which exceeds the required sample size and ensures the robustness of the study’s findings.

A team of 20 volunteer data collectors was set up, such that 2 volunteers (a male and female) were randomly selected from each of the 10 universities. The volunteers were trained on data collection procedures and did not participate in filling the survey. The participants of this study were approached and recruited through their various class WhatsApp group chats (1st year to 6th year), and their NOSA chapter WhatsApp group, as this is the platform where the majority of students get access to their academic information and other relevant announcements. The study participants were all literate, as only optometry students were involved in the study, and in terms of age minority, none of the participants were below the age of 15.

### Data collection

The questionnaire used in this study was adapted from validated instruments previously employed in similar studies conducted in South Africa [8], Ghana [9], and India [10]. Prior to data collection, a pilot study was conducted with 10 students to assess the clarity and reliability of the questionnaire and research procedures. Participants were asked to complete the questionnaire twice, with a two-week interval between each session. The consistency of responses was analyzed, and any identified issues were resolved by revising ambiguous or unclear questions. The final version of the questionnaire was administered online using Google Forms, which was shared via WhatsApp (group chats) – which is the most widely used communication channel among Nigerian optometry students.

The administered questionnaire evaluated their demographic attributes, opinions regarding practice preferences, and the factors that would impact their choice to work in rural areas. Data collection was supported by a group of trained volunteer optometry students selected across the ten universities using the official NOSA WhatsApp group(s). The sample size chosen was sufficiently large to effectively represent the study population and was consistent with a previously conducted study [16]. Participants were encouraged to provide candid responses, with the assurance that their identities would be kept confidential.

In the context of this research, a rural region is defined as a geographic region or community that is located outside urban centres and is characterized by a low population density (< 20,000 inhabitants), limited infrastructure, and a predominantly agricultural or natural resource-based economy [19]. An urban area refers to a geographic region or settlement characterized by a high population density (> 20,000 inhabitants), extensive built-up infrastructure, and diverse economic activities [19].

**Table 1:** Demographics of Nigerian optometry students (*n* = 400)

	Year													
	100 Level		200 Level		300 Level		400 Level		500 Level		600 Level		Total	
	*N*	%	*N*	%	*N*	%	*N*	%	*N*	%	*N*	%	*N*	%
**Age**														
15–19	59	78.7	21	42.0	14	25.9	6	8.1	0	0.0	0	0.0	100	25.0
20–24	16	21.3	29	58.0	36	66.7	58	78.4	38	84.4	61	59.8	238	59.5
25–29	0	0.0	0	0.0	3	5.6	9	12.2	7	15.6	39	38.2	58	14.5
30–34	0	0.0	0	0.0	0	0.0	1	1.4	0	0.0	1	1.0	2	0.5
35–40	0	0.0	0	0.0	1	1.9	0	0.0	0	0.0	1	1.0	2	0.5
>=41	0	0.0	0	0.0	0	0.0	0	0.0	0	0.0	0	0.0	0	0.0
**Gender**														
Male	25	33.3	12	24.0	12	22.2	24	32.4	16	35.6	29	28.4	118	29.5
Female	50	66.7	38	76.0	42	77.8	50	67.6	29	64.4	73	71.6	282	70.5
**Marital Status**														
Single	75	100.0	50	100.0	53	98.1	71	95.9	44	97.8	95	93.1	388	97.0
Married	0	0.0	0	0.0	1	1.9	3	4.1	1	2.2	7	6.9	12	3.0
**Institution**														
ABSU	0	0.0	1	2.0	4	7.4	6	8.1	7	15.6	14	13.7	32	8.0
ABUAD	1	1.3	0	0.0	1	1.9	3	4.1	2	4.4	0	0.0	7	1.8
BUK	0	0.0	4	8.0	0	0.0	9	12.2	2	4.4	8	7.8	23	5.8
BU	10	13.3	5	10.0	13	24.1	4	5.4	1	2.2	0	0.0	33	8.3
FUTO	0	0.0	4	8.0	3	5.6	4	5.4	1	2.2	1	1.0	13	3.3
IMSU	1	1.3	0	0.0	0	0.0	2	2.7	0	0.0	8	7.8	11	2.8
MUE	0	0.0	2	4.0	6	11.1	6	8.1	2	4.4	7	6.9	23	5.8
NU	0	0.0	3	6.0	3	5.6	2	2.7	0	0.0	0	0.0	8	2.0
UNIBEN	43	57.3	23	46.0	15	27.8	31	41.9	25	55.6	60	58.8	197	49.3
University of Ilorin, Ilorin	20	26.7	8	16.0	9	16.7	7	9.5	5	11.1	4	3.9	53	13.3
**Year**														
100 Level	75	100.0	0	0.0	0	0.0	0	0.0	0	0.0	0	0.0	75	18.8
200 Level	0	0.0	50	100.0	0	0.0	0	0.0	0	0.0	0	0.0	50	12.5
300 Level	0	0.0	0	0.0	54	100.0	0	0.0	0	0.0	0	0.0	54	13.5
400 Level	0	0.0	0	0.0	0	0.0	74	100.0	0	0.0	0	0.0	74	18.5
500 Level	0	0.0	0	0.0	0	0.0	0	0.0	45	100.0	0	0.0	45	11.3
600 Level	0	0.0	0	0.0	0	0.0	0	0.0	0	0.0	102	100.0	102	25.5
**Place**														
Rural	22	29.3	15	30.0	20	37.0	20	27.0	15	33.3	31	30.4	123	30.8
Urban	53	70.7	35	70.0	34	63.0	54	73.0	30	66.7	71	69.6	277	69.2
**Region**														
North-East	1	1.3	1	2.0	3	5.6	1	1.4	0	0.0	1	1.0	7	1.8
North-West	1	1.3	5	10.0	3	5.6	7	9.5	3	6.7	6	5.9	25	6.3
North-Central	23	30.7	7	14.0	9	16.7	8	10.8	4	8.9	4	3.9	55	13.8
South-East	5	6.7	9	18.0	17	31.5	19	25.7	14	31.1	33	32.4	97	24.3
South-West	12	16.0	12	24.0	8	14.8	11	14.9	4	8.9	11	10.8	58	14.5
South-South	33	44.0	16	32.0	14	25.9	28	37.8	20	44.4	47	46.1	158	39.5

### Data analysis

The Statistical Program for Social Sciences (SPSS), version 25.0, was used to collect and analyse the data. Descriptive statistics such as frequencies, mean, standard deviation, percentages, and cross-tabulations was used. Chi-square tests was used to determine relationships between relevant variables. Statistical significance was recognized when the *p*-value was less than 0.05.

The data was stratified based on several demographic and academic variables, including year of study, geographic origin (urban or rural), and other socio-demographic factors. This stratification was employed to explore trends and differences in students’ preferences and motivations across different subgroups. Specifically, stratified analyses allowed for a more detailed examination of how factors such as urban versus rural upbringing or academic seniority (first-year through sixth-year) influenced students’ decisions regarding rural optometric practice. The results of these stratified analyses are presented in Tables 2, 3, 4, 5, 6 and 7.

**Table 2:** Optometry students’ view about practice choice (100 level, *n* = 75)

Variables		Rural		Urban		Total	
		*N*	%	*N*	%	*N*	%
**Do you intend to establish your first optometric practice in the rural?**	Yes	5	26.3	14	73.7	19	25.3
	No	17	30.4	39	69.6	56	74.7
**Do you intend to establish your subsequent optometric practice in the rural area?**	Yes	14	32.6	29	67.4	43	57.3
	No	8	25.0	24	75.0	32	42.7
**Do you intend to be employed by the government to work in a rural?**	Yes	3	33.3	6	66.7	9	12.0
	No	19	28.8	47	71.2	66	88.0
**Do you intend to be employed by an NGO to work in a rural area?**	Yes	8	28.6	20	71.4	28	37.3
	No	14	29.8	33	70.2	47	62.7
**After graduation**,** should there be a compulsory posting of optometrists to rural areas by the government?**	Yes	10	27.0	27	73.0	37	49.3
	No	12	31.6	26	68.4	38	38.7

**Table 3:** Optometry students’ view about practice choice (200 level, *n* = 50)

Variables		Rural		Urban		Total	
		*N*	%	*N*	%	*N*	%
**Do you intend to establish your first optometric practice in the rural?**	Yes	7	41.2	10	58.8	17	34.0
	No	8	24.2	25	75.8	33	66.0
**Do you intend to establish your subsequent optometric practice in the rural area?**	Yes	10	29.4	24	70.6	34	68.0
	No	5	31.3	11	68.8	16	32.0
**Do you intend to be employed by the government to work in a rural?**	Yes	6	35.3	11	64.7	17	34.0
	No	9	27.3	24	72.7	33	66.0
**Do you intend to be employed by an NGO to work in a rural area?**	Yes	8	34.8	15	65.2	23	46.0
	No	7	25.9	20	74.1	27	54.0
**After graduation**,** should there be a compulsory posting of optometrists to rural areas by the government?**	Yes	7	26.9	19	73.1	26	52.0
	No	8	33.3	16	66.7	24	48.0

**Table 4:** Optometry students’ view about practice choice (300 level, *n* = 54)

Variables		Rural		Urban		Total	
		*N*	%	*N*	%	*N*	%
**Do you intend to establish your first optometric practice in the rural?**	Yes	4	33.3	8	66.7	12	22.2
	No	16	38.1	26	61.9	42	77.8
**Do you intend to establish your subsequent optometric practice in the rural area?**	Yes	11	36.7	19	63.3	30	55.6
	No	9	37.5	15	62.5	24	44.4
**Do you intend to be employed by the government to work in a rural?**	Yes	7	50.0	7	50.0	14	26.0
	No	13	32.5	27	67.5	40	70.0
**Do you intend to be employed by an NGO to work in a rural area?**	Yes	9	37.5	15	62.5	24	44.4
	No	11	36.7	19	63.3	30	55.6
**After graduation**,** should there be a compulsory posting of optometrists to rural areas by the government?**	Yes	8	47.1	9	52.9	17	31.5
	No	12	32.4	25	67.6	37	68.5

**Table 5:** Optometry students’ view about practice choice (400 level, *n* = 74)

Variables		Rural		Urban		Total	
		*N*	%	*N*	%	*N*	%
**Do you intend to establish your first optometric practice in the rural?**	Yes	4	40.0	6	60.0	10	13.5
	No	16	25.0	48	75.0	64	86.5
**Do you intend to establish your subsequent optometric practice in the rural area?**	Yes	9	20.5	35	79.5	44	59.5
	No	11	36.7	19	63.3	30	40.5
**Do you intend to be employed by the government to work in a rural?**	Yes	1	7.7	12	92.3	13	17.6
	No	19	31.1	42	68.9	61	82.4
**Do you intend to be employed by an NGO to work in a rural area?**	Yes	9	25.7	26	74.3	35	47.3
	No	11	28.2	28	71.8	39	52.7
**After graduation**,** should there be a compulsory posting of optometrists to rural areas by the government?**	Yes	11	39.3	17	60.7	28	37.8
	No	9	19.6	37	80.4	46	62.2

**Table 6:** Optometry Students’ View about Practice Choice (500 level, *n* = 45)

Variables		Rural		Urban		Total	
		*N*	%	*N*	%	*N*	%
**Do you intend to establish your first optometric practice in the rural?**	Yes	1	33.3	2	66.7	3	6.7
	No	14	33.3	28	66.7	42	93.3
**Do you intend to establish your subsequent optometric practice in the rural area?**	Yes	6	31.6	13	68.4	19	42.2
	No	9	34.6	17	65.4	26	57.8
**Do you intend to be employed by the government to work in a rural?**	Yes	4	28.6	10	71.4	14	31.1
	No	11	35.5	20	64.5	31	68.9
**Do you intend to be employed by an NGO to work in a rural area?**	Yes	6	26.1	17	73.9	23	51.1
	No	9	40.9	13	59.1	22	48.9
**After graduation**,** should there be a compulsory posting of optometrists to rural areas by the government?**	Yes	7	43.8	9	56.3	16	35.6
	No	8	27.6	21	72.4	29	64.4

**Table 7:** Optometry Students’ View about Practice Choice (600 level, *n* = 102)

Variables		Rural		Urban		Total	
		*N*	%	*N*	%	*N*	%
**Do you intend to establish your first optometric practice in the rural?**	Yes	5	35.7	9	64.3	14	13.7
	No	26	29.5	62	70.5	88	86.3
**Do you intend to establish your subsequent optometric practice in the rural area?**	Yes	12	29.3	29	70.7	41	40.2
	No	19	31.1	42	68.9	61	59.8
**Do you intend to be employed by the government to work in a rural?**	Yes	10	37.0	17	63.0	27	26.5
	No	21	28.0	54	72.0	75	73.5
**Do you intend to be employed by an NGO to work in a rural area?**	Yes	16	35.6	29	64.4	45	44.1
	No	15	26.3	42	73.7	57	55.9
**After graduation**,** should there be a compulsory posting of optometrists to rural areas by the government?**	Yes	13	36.1	23	63.9	36	35.3
	No	18	27.3	48	72.7	66	64.7

### Ethical consideration

Ethical approval to conduct this study was obtained from the Research and Ethics Committee of the Department of Optometry, University of Benin. The research followed the principles outlined in the Helsinki Declaration, and only students who provided informed consent were included in the study. In order to preserve anonymity, no personal information was gathered from the participants. At the beginning of the research survey, participants were presented with a detailed information page outlining the purpose of the study, potential risks and benefits, and the voluntary nature of participation. Prior to proceeding with the survey questions, participants were explicitly informed that their voluntary completion of the survey implies their consent to participate. Only those who acknowledged understanding this information and chose to proceed were included in the study. This informed consent process adheres to ethical standards for online research.

## Results

### Demographic attributes

400 Nigerian optometry students participated in this study. The study consisted of 70.5% females and 29.5% males between the ages of 20–24 years (mean 21.60 years ± 3.38). There was a very high number of unmarried people (*n* = 388; 97.0%) with University of Benin constituting most of the students (*n* = 197; 49.3%). Majority of the participants were in their final year (600 Level) of study (*n* = 102; 25.5%), most were from urban areas (*n* = 277; 69.3%), and the majority were from the South-South region of Nigeria (*n* = 158, 39.5%). Detailed socio-demographic information about the study participants can be found in Table 1.

## Characteristics of optometry students from rural areas

**Fig. 2:**
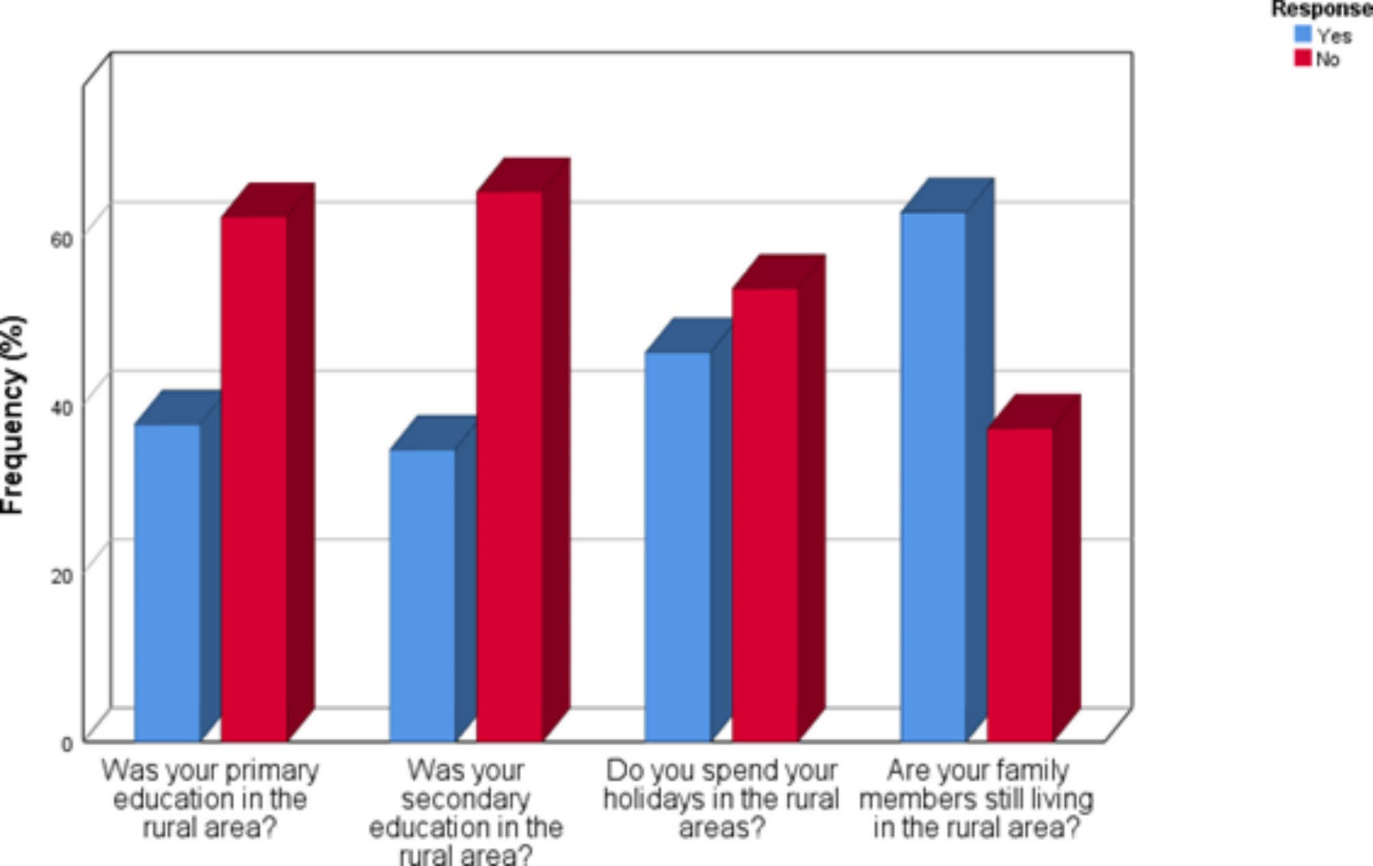
Characteristics of respondents from rural areas

Figure 2 shows that the majority of optometry students attended their primary (*n* = 76; 62.3%) and secondary schools (*n* = 79; 65.3%) in urban areas. Most respondents from rural areas do not spend their holidays in the rural region (*n* = 64; 53.8%), however, most of them have family members living in rural areas. (*n* = 76; 62.8%).

### Optometry students’ view about practice choice

A significant portion (81.3%, *n* = 325) of the respondents indicated that they do not intend to establish their first optometric practice in rural areas (Table 2). This reluctance was predominantly observed among students from urban backgrounds (70.2%, *n* = 228), with no significant difference observed when compared to students from rural backgrounds (29.8%, *n* = 97; *p* = 0.415).

However, most would consider to establish their subsequent practices in the rural area (*n* = 211; 52.8%), of which majority were from urban areas (*n* = 149; 70.6%). Overall, the majority of the participants indicated their reluctance to accept postings to rural areas by the government (*n* = 306; 76.5%) or NGOs (*n* = 222; 55.5%). Also, a considerable number of participants (*n* = 240; 60%), of which majority were students from urban background (*n* = 173; 72.1%), opposed compulsory posting to rural areas.

The results of students’ views about working in rural areas was stratified across all 6 student classes (100–600 level) in Tables 2, 3, 4, 5, 6 and 7. To address the imbalance between respondents from urban (69.3%, *n* = 277) and rural (30.8%, *n* = 123) areas, chi-square tests were used to assess the relationship between place of origin and preferences for rural practice. The analysis showed no statistically significant difference (*p* > 0.05, Table 8) between urban and rural students’ likelihood of choosing rural practice, ensuring that the imbalance did not bias the results.

**Table 8:** Odds for students from urban background to reject working in rural areas in comparison with those of rural background

	Odd Ratio	95% Confidence Interval		*P*-Value
		Lower	Upper	
**Establish your first optometric practice in the rural area**	1.247	0.733	2.122	0.415
**Establish your subsequent optometric practice in the rural area**	0.873	0.571	1.336	0.532
**Employed by the government to work in a rural area**	1.145	0.698	1.877	0.592
**Employed by an NGO to work in a rural area**	1.062	0.693	1.627	0.783
**Compulsory posting of optometrists to rural areas by the government?**	1.390	0.904	2.138	0.133

These tables present the percentage of students (in each undergraduate level) from rural and urban backgrounds who expressed preferences regarding establishing their first optometric practice in rural areas, subsequent practice preferences, and willingness to accept rural postings by the government or NGOs.

### Motivation for selecting rural practice

**Fig. 3:**
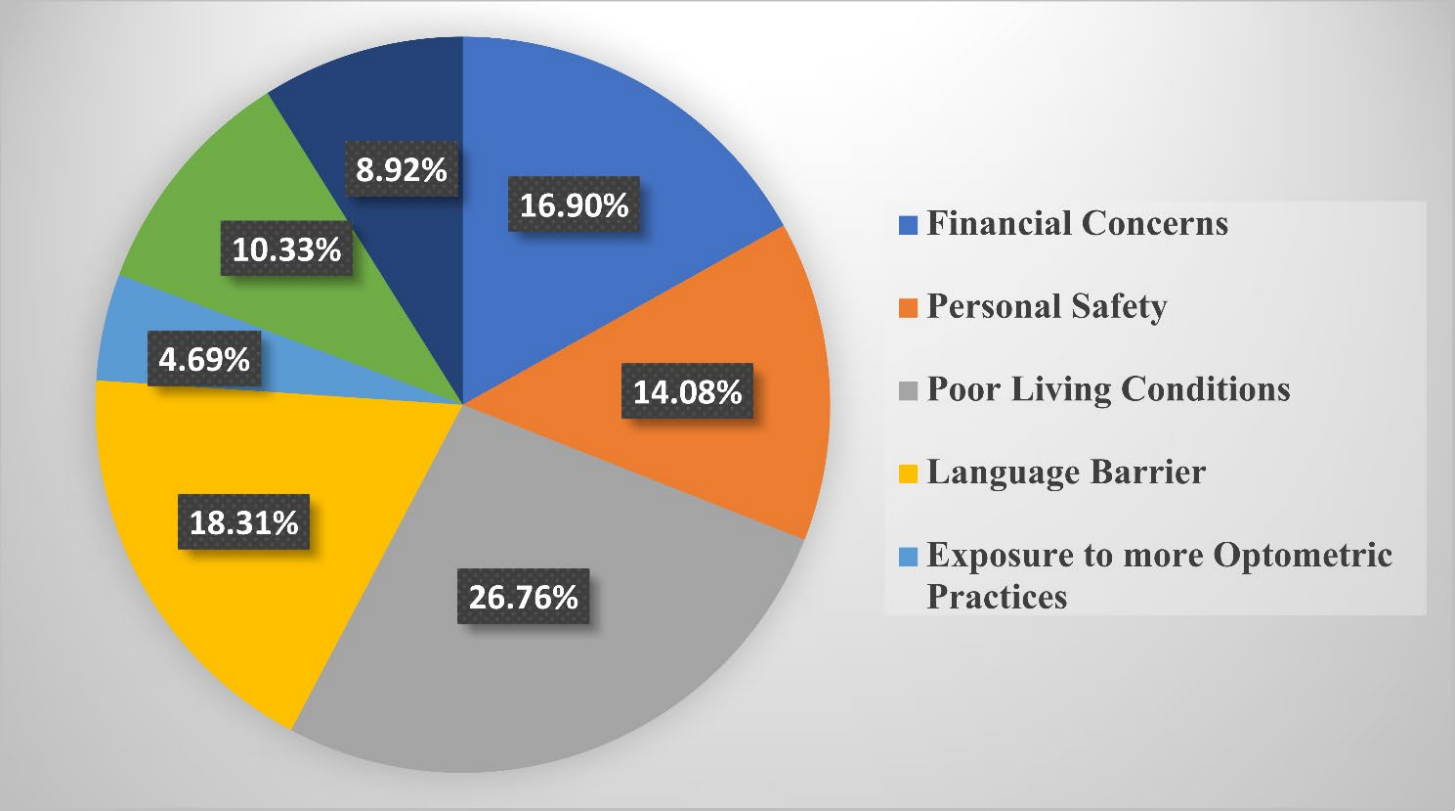
Factors that would be considered by 100 level students when deciding on rural practice

**Fig. 4:**
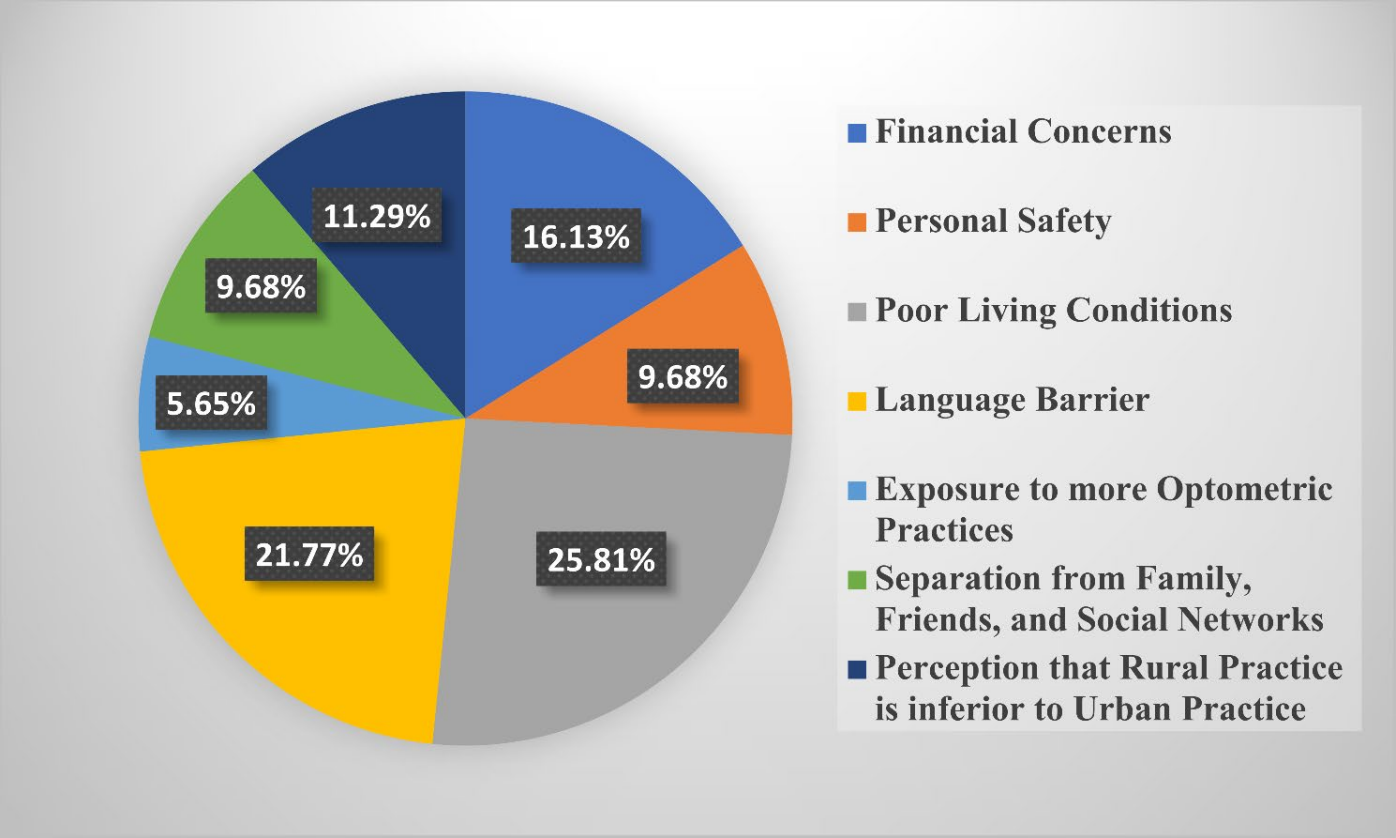
Factors that would be considered by 200 level students when deciding on rural practice

**Fig. 5:**
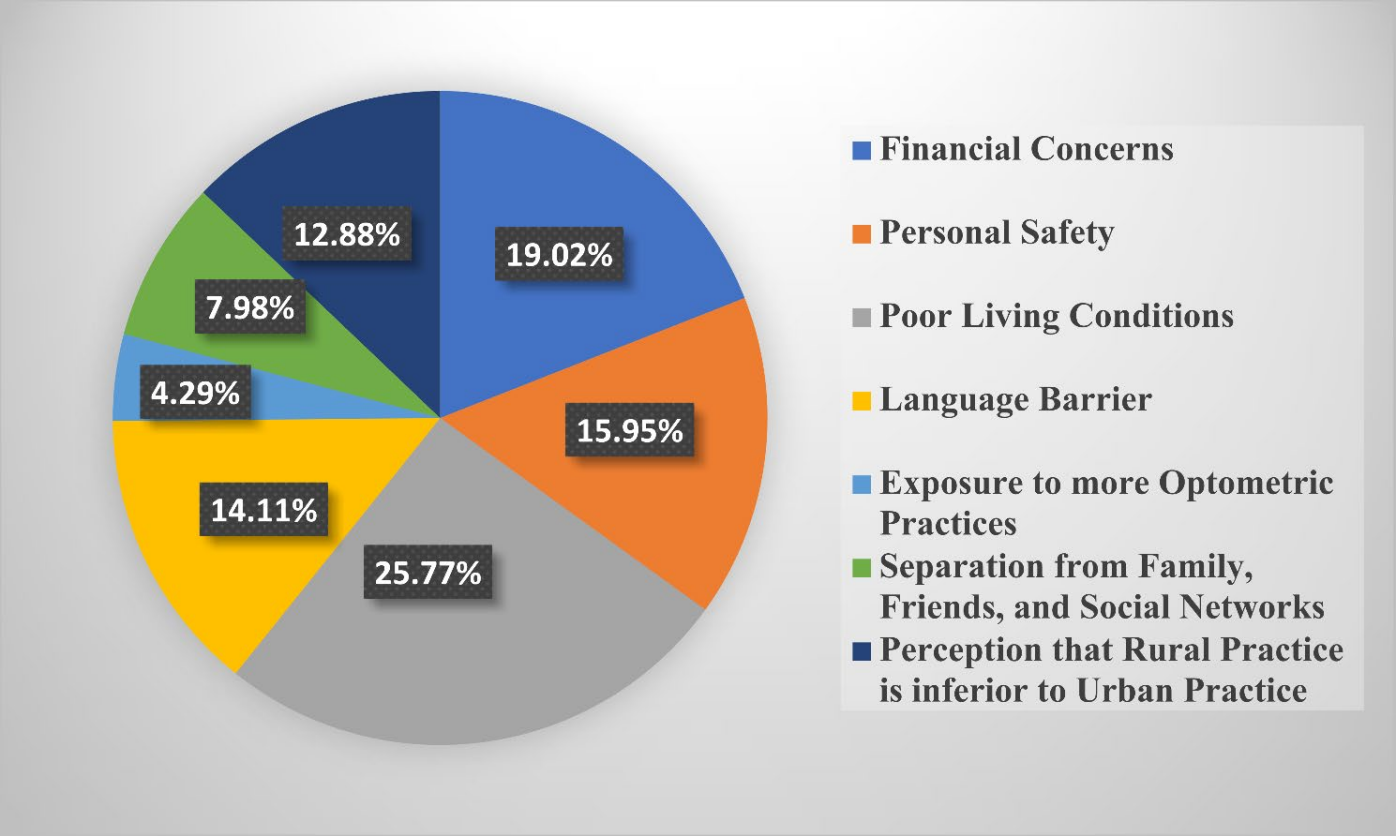
Factors that would be considered by 300 level students when deciding on rural practice

**Fig. 6:**
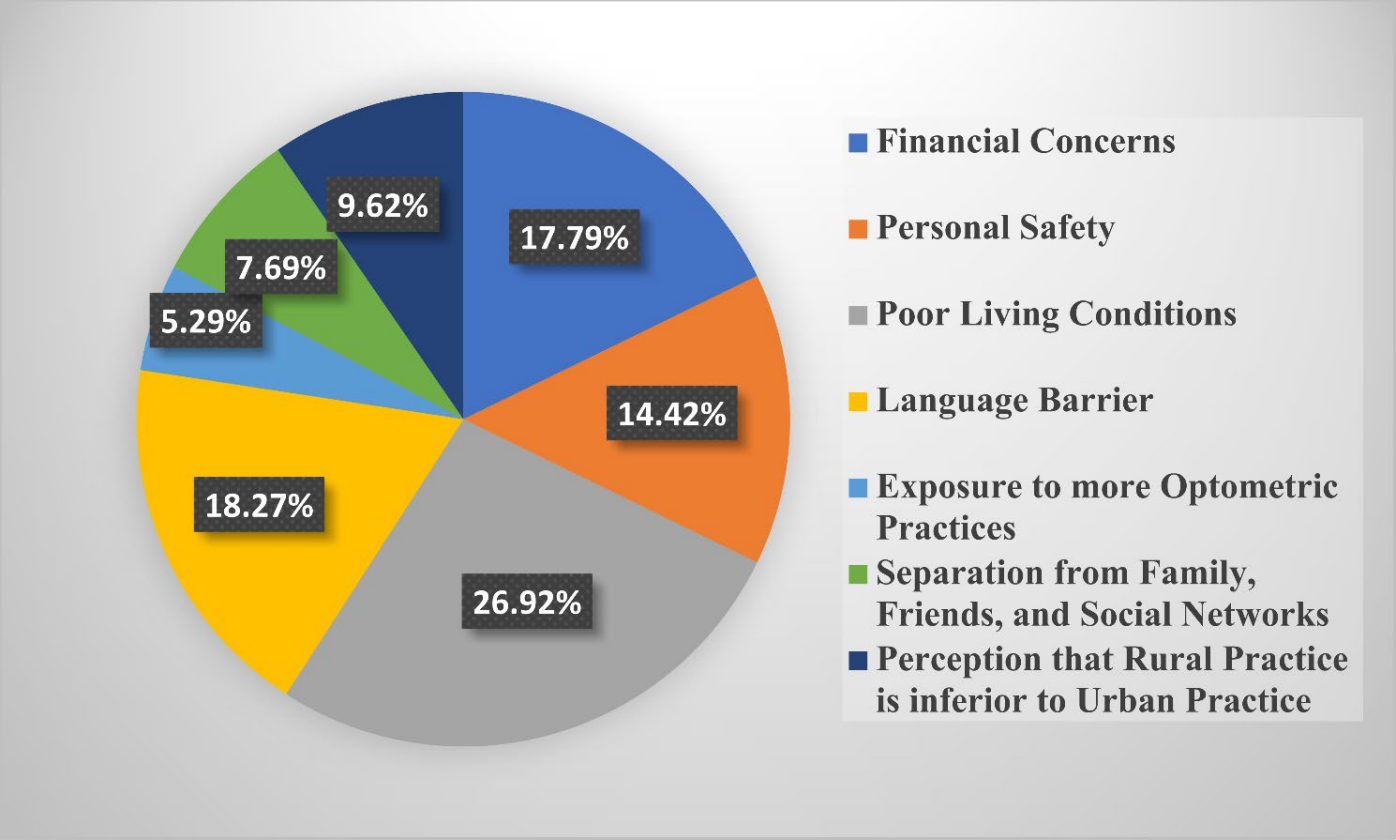
Factors that would be considered by 400 level students when deciding on rural practice

**Fig. 7:**
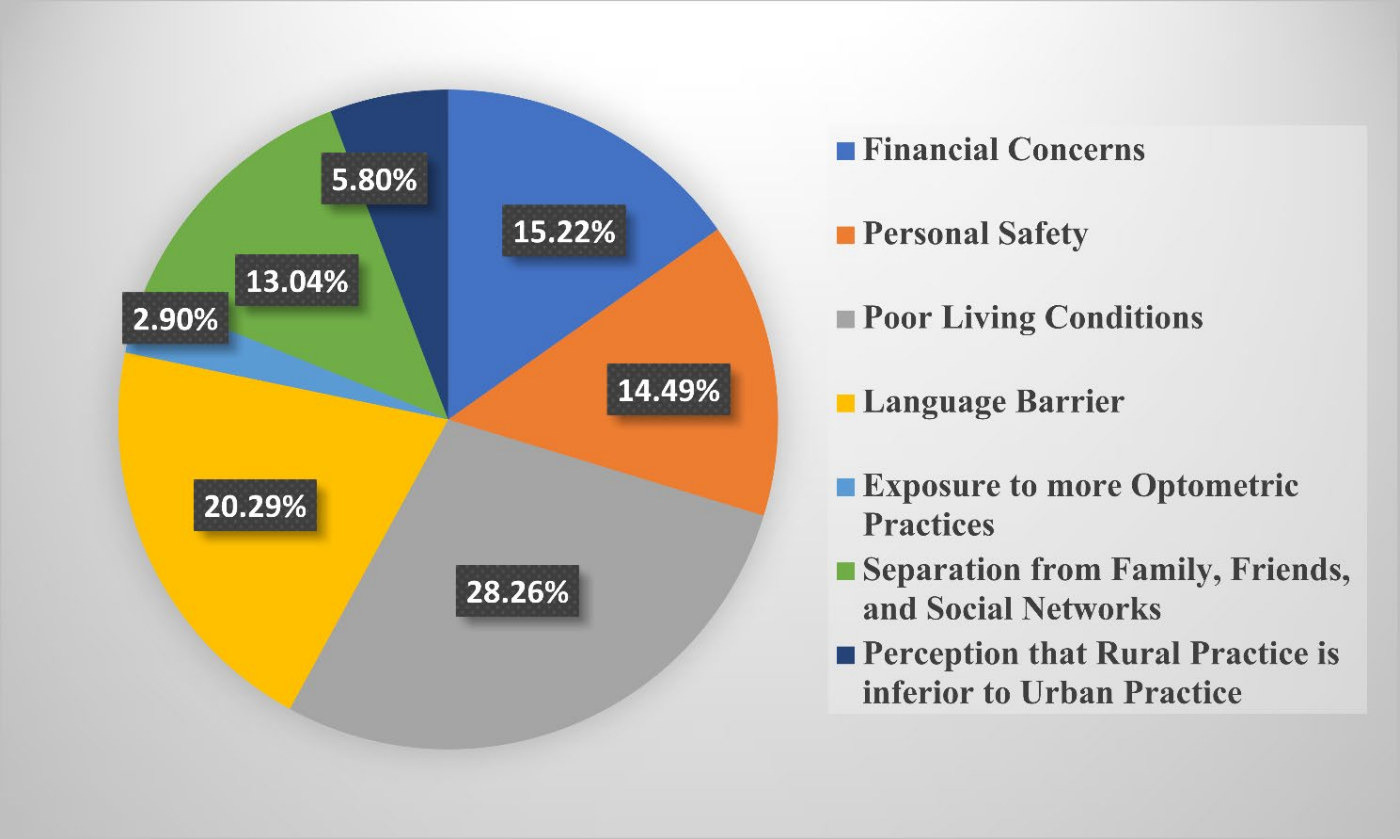
Factors that would be considered by 500 level students when deciding on rural practice

**Fig. 8:**
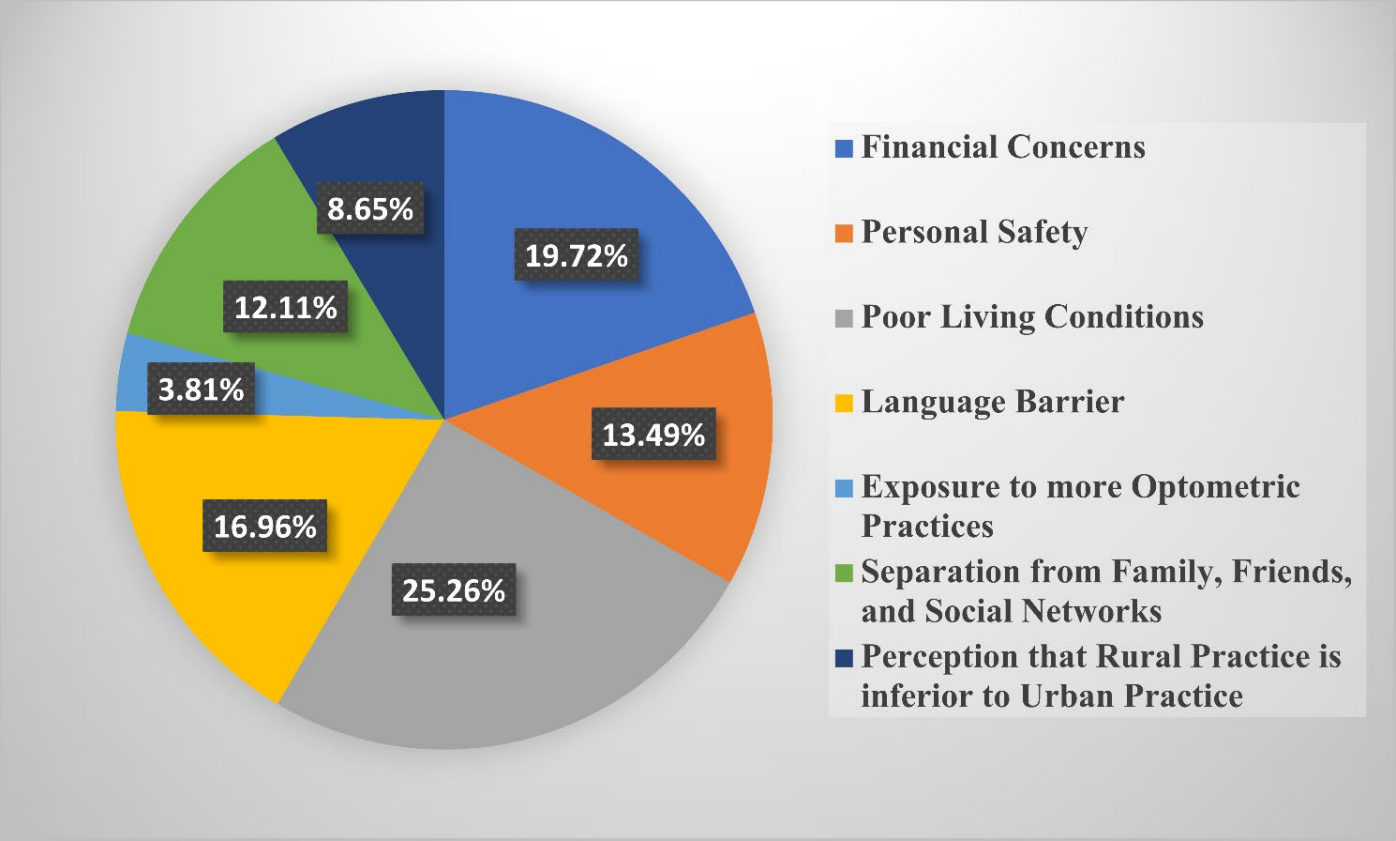
Factors that would be considered by 600 level students when deciding on rural practice

The barriers to choosing rural practice identified by respondents were primarily poor living conditions (26.34%, *n* = 299), language barriers (17.97%, *n* = 204), and financial concerns (17.8%, *n* = 202). Other factors included personal safety concerns and limited professional development opportunities, which were highlighted in qualitative feedback. These barriers were consistent across all student levels and were found to significantly influence the decision to reject rural practice. The results of students’ perspectives about the factors they would consider in deciding on rural practice was stratified across all 6 student classes (100–600 level) in Figs. 3, 4, 5, 6, 7 and 8. The motivating factors for choosing to work in rural areas was presented in Table 9. It shows that the majority of the participants would be motivated to work in a rural area to ‘help the community’ (*n* = 334; 56.6%). The least motivating factor was ‘proximity to their home’ (*n* = 32; 5.4%), closely accompanied by ‘inability to find jobs’ (*n* = 42; 7.1%).

**Table 9:** Motivating factors for choosing to work in the rural area

Motivation	*N*	Percent %
**Good Salary**	100	16.9
**Proximity to Home**	32	5.4
**Inability to Find a Job**	42	7.1
**Help the Community**	334	56.6
**Extra Incentives**	82	13.9
**Total**	590	100.0

### Factors that influence optometry students’ decision to engage in rural practice

A significant proportion of the participants indicated that enhancement of optometric practice (*n* = 298; 74.6%), outreach interaction between rural and urban health workers (*n* = 265; 66.3%) and opportunities for professional development (*n* = 255; 63.8%) were the most appealing incentives that will encourage them to work in rural areas. This is outlined in Table 10 below.

**Table 10:** Factors that influence optometry students’ decision to engage in rural practice

Level of Attractiveness						
Incentives	VA (%)	MA (%)	SA (%)	NRA (%)	NAA (%)	MA+ VA (%)
**Opportunities for professional development**	132(33.15)	123(30.65)	54(13.5)	62(15.5)	29(7.2)	255(63.8)
**Scholarship and bursary for further studies**	173(43.3)	77(19.3)	54(13.5)	60(15.0)	36(9.0)	250(62.6)
**Conducive or favourable working environment**	164(41.05)	78(19.55)	60(15.0)	49(12.3)	49(12.3)	242(60.6)
**Financial incentives**	152(38.05)	94(23.55)	54(13.5)	62(15.5)	38(9.5)	246(61.6)
**Better living conditions**	169(42.4)	76(18.9)	35(8.8)	70(17.5)	50(12.5)	245(61.3)
**Access to free/subsidized Continuous Professional Development (CPD) courses**	153(38.45)	87(21.65)	70(17.5)	58(14.5)	32(8.0)	240(60.1)
**Outreach interaction between rural and urban health workers**	154(38.55)	111(27.75)	80(20.0)	41(10.3)	14(3.5)	265(66.3)
**Enhance scope of optometric practice**	195(48.8)	103(25.8)	49(12.3)	31(7.8)	22(5.5)	298(74.6)
**Compulsory community service**	90(22.5)	116(29)	90(22.5)	71(17.8)	33(8.3)	206(51.5)

VA = Very Attractive; MA = Moderately Attractive; SA = Slightly Attractive; NRA = Not Really Attractive; NAA = Not Attractive At All, and MA + VA = Moderately Attractive and Very Attractive

### Influence of demographic variables on students’ choice of rural practice

In addition to descriptive statistics, chi-square test was performed to determine the influence of demographic variables like gender, year of study and place of origin, on students’ choice of rural practice. The results of the chi-square test shown in Table 11 revealed that there was a significant relationship between participants’ year of study and their preference to practice in rural areas (*p* < 0.05). However, there was no significant relationship between participants’ gender and place of origin, and their preferences to practice in rural areas (*p* > 0.05). Therefore, while neither gender nor place of origin has any significant effect on the preference for rural practice, the year of study does play a significant role.

**Table 11:** Chi-square test for demographic variables and students’ preference

		Rural Practice		Total	Chi-Square *P*-value
		Yes	No		
**Gender**	Male	29	89	118	0.053
	Female	46	236	282	
Total		75	325	400	
**Year**	100 Level	19	56	75	0.004
	200 Level	17	33	50	
	300 Level	12	42	54	
	400 Level	10	64	74	
	500 Level	3	42	45	
	600 Level	14	88	102	
Total		75	325	400	
**Place**	Rural	26	97	123	0.415
	Urban	49	228	277	
Total		75	325	400	

## Discussion

In many developing nations, including Nigeria, there exists an imbalanced distribution of eye healthcare professionals, which disproportionately affects rural areas [9]. This is primarily due to the concentration of a majority of eye care practitioners in urban areas, leaving rural residents, who often require their services the most, underserved [7, 13]. Therefore, this study showed the factors influencing the choice of practice location among optometry students in Nigeria.

The study showed that most of the respondents (81.3%) who, in line with the findings of [8] (66%) and [9] (65.2%), expressed reluctance to initiate their first practice in rural areas. Conversely, Mondal et al. [10] reported that 64.2% of participants were open to considering the establishment of their inaugural practice in rural regions, presenting a contrasting viewpoint. This could be attributed to the fact that the majority of the optometry students from the Indian study were from rural backgrounds, and felt they were in a better position to understand their community’s eye care needs. Hence, they were more inclined to work in their place of origin.

The studies by Mashige et al. [8]. and Boadi-Kusi et al. [9]. are comparable to this study (mean 21.60 years ± 3.38) in terms of similar mean age (21.1 ± 1.8 and 22.67 ± 2.03 respectively) of respondents. However, it diverges in terms of gender distribution, with a larger proportion of female participants (70.5%) in this study compared to the Ghanaian [9] study (26.4%) but similar to the South African [8] (67%) and Indian [10] study (52%). This increase in the representation of female optometry students is consistent with a previous investigation into the gender distribution of eye care workforce in Nigeria, which shows that there are more females interested in becoming an optometrist, and more female eye care workers (86%) were concentrated in urban regions compared to rural regions [3]. Also, another study recognised that women are less prone to accept rural posts and are underrepresented in rural areas [20]. In light of this, the fact that the sample population in this study had more representation of females may have had an effect on their preference of urban practice over rural practice. In addition, the results of the chi-square test showed that the year of study of the participants had a significant influence on their choice of rural or urban practice, while on the other hand, neither gender nor place of origin had any significant role to play.

Furthermore, a significant portion of students in diverse medical programs, such as optometry, frequently come from urban backgrounds [8, 21–23]. These students generally exhibit a reluctance toward seeking employment opportunities in rural settings. Similarly, findings from studies conducted [24–27] suggest that respondents were less interested in rural employment, as rural areas often lack the infrastructural facilities required for adequate medical practice. This pattern is mirrored among optometry students in Nigeria, affirming the notion that students in healthcare programs often gravitate toward urban practice. Unfortunately, this trend exacerbates the issue of restricted healthcare availability in rural areas.

In Nigeria, government agencies and NGOs serve as the primary sources of healthcare provision, especially in rural regions. However, this study indicates that a significant majority of Nigerian optometry students, particularly those with urban backgrounds, would decline offers of rural jobs or postings from government and NGOs. This finding contradicts the results reported by Mashige et al. [8]. , Boadi-Kusi et al. [9]. , Mondal et al. [10]. and Gonzaga et al. [28]. One possible explanation for this difference may be rooted in the rising insecurity issues in Nigeria that causes a decline in rural healthcare workforce, as highlighted in previous reports [29–31]. The current study also shows that there was no statistically significant difference (*p* > 0.05) between the likelihood of students from urban backgrounds to choose to work in rural areas compared to students from rural backgrounds. This inclination might be attributed to the deeper understanding and firsthand experience that rural-background students possess regarding the eye care challenges faced by their communities [32, 33].

Poor living conditions emerged as the primary consideration for Nigerian optometry students when contemplating rural practice. This finding is consistent with other previous studies [25, 34] that recognized unfavourable living conditions as one of the hindrances in rural medical practice. This may be as a result of inadequate fundamental services and infrastructural healthcare facilities in rural areas [35, 36]. However, financial concerns have been identified as a significant obstacle to practice in the rural areas, as revealed in similar previous studies that involve optometry students [8–10]. Moreover, the majority of respondents in this study expressed that their motivation of choosing to work in rural areas is based on ‘helping the community’ (*n* = 334; 56.6%). This aligns with previous studies [10, 23, 27, 37–39] that affirms that students’ desire to help their community was the major motivational factor to practice in rural areas. Interestingly, this contradicts the findings [8, 9] where a good salary was recognized as the primary motivating factor for Ghanaian and South African optometry students, respectively.

Likewise, the strong opposition to rural optometric practice could be attributed to the absence of certain incentives and attractions. They include enhancement of optometric practice (74.6%), outreach interaction between rural and urban health workers (66.3%), and opportunities for professional development (63.8%). These incentives were identified as the most compelling factors that would encourage optometry students to pursue rural practice (post-graduation). Unlike Mashige et al. [8]. that had a favourable working environment, Boadi-Kusi et al. [9]. and Gonzaga et al. [28]. had financial incentives, and Mondal et al.. (2021) was scholarship for further studies. This could be as a result of the zeal for many Nigerian students to get better in their career [40]. It is vital to underscore that the implementation of initiatives focused on these aspects would not only draw more optometry students toward rural practice after completing their education but also improve the availability and quality of eye care services in underserved areas.

While this study provides valuable insights, it acknowledges some limitations that may affect the generalizability of the findings. The use of a convenience sampling technique, while practical for assessing a large number of participants, presents potential limitations. As participants were recruited through WhatsApp group chats of registered students, this may introduce selection bias. Students who were more active in these groups may have been more likely to participate, potentially skewing the sample toward more engaged students. Additionally, although a total of 400 participants was obtained, however, the study was concentrated in certain universities which was due to the fact that some universities were more populated than others, and these universities specifically offered the O.D. program, omitting those that either lack full accreditation or are in the process of obtaining it. Also, the sample size appears to be skewed, particularly towards Benin city, which limits the broader applicability of the study’s findings to Nigeria as a whole. The absence of systematic sampling is a notable factor contributing to this skewness. Implementing systematic sampling would have been instrumental in ensuring a more balanced and representative inclusion of participants from various regions within Nigeria, in order to make comprehensive assertions about the optometry landscape across the country.

Another potential limitation of this study is the reliance on self-reported data. Participants’ responses may be influenced by social desirability bias, particularly in questions related to their willingness to work in rural areas. Students may have provided responses they believe are expected or socially acceptable rather than reflecting their true intentions. Another factor that might have influenced participant responses is the economic background of the students, and how they financed their education, which was not examined in this study. Future studies are necessary to explore such additional factors and expand the participant base across a wider range of institutions to further validate these findings.

Certain approaches can be employed to compel more optometry students and optometrists toward rural practice. Firstly, it is of utmost importance to improve rural infrastructure through strategic budgetary allocations from the national and state arms of government, aimed at upgrading rural clinics, providing modern medical equipment, and improving living conditions such as housing, electricity, and water supply [41]. Furthermore, rotational programs can be implemented such that optometry students are posted rotationally between rural and urban areas, to expose them to the realities of rural healthcare while balancing their desire for urban amenities. A similar approach is evident in the Community Health Nursing module currently implemented in the Nursing science undergraduate program in Nigeria, where Nursing students undergo rotations in rural communities, in addition to their clinical rotations in urban hospitals [42]. Modifications to the Optometry undergraduate program can be modelled after this, and optimised to suit the program curriculum. Furthermore, financial incentives can be harnessed to attract healthcare professionals to rural areas. For instance, in the case of South Africa, a rural allowance is provided to medical professionals who agree to work in underserved areas. This allowance, in combination with housing and other benefits, has contributed to a modest increase in rural health workforce retention [43]. A similar system could be implemented among optometrists in Nigeria to reduce hesitancy towards rural practice.

## Conclusion

This study reveals that most optometry students in Nigeria, particularly those from urban backgrounds, are reluctant to practice in rural areas after graduation, primarily due to concerns over living conditions. In contrast, students from rural backgrounds show a higher likelihood of considering rural practice, especially within NGOs or the public sector. The key motivation for all students, regardless of background, was the opportunity to help their community. Based on these findings, we recommend targeted interventions to enhance rural practice engagement, such as offering tailored incentives for optometrists willing to serve in rural areas, especially those aimed at improving living and working conditions. Pilot programs to promote rural placements among optometry students should also be explored. These steps could be more feasible than broad policy changes.

## Data Availability

No datasets were generated or analysed during the current study.

## Abbreviations

O.D: Doctor of Optometry
NGO: Non-governmental organization
WHO: World Health Organization
UNIBEN: University of Benin
UNILORIN: University of Ilorin
BUK: Bayero University, Kano
ABSU: Abia State University
IMSU: Imo State University
FUTO: Federal University of Technology, Owerri
BU: Bingham University
ABUAD: Afe Babalola University, Ado-Ekiti
NU: Novena University
MUE: Madonna University, Elele
NOSA: Nigerian Optometric Students’ Association
